# Characterization of receptor tyrosine kinase activation and biological activity of toceranib phosphate in canine urothelial carcinoma cell lines

**DOI:** 10.1186/s12917-021-03027-0

**Published:** 2021-10-02

**Authors:** Daniela I. Korec, Darian S. Louke, Justin T. Breitbach, Jennifer A. Geisler, Brian D. Husbands, Joelle M. Fenger

**Affiliations:** 1 Department of Veterinary Clinical Sciences, College of Veterinary Medicine, The Ohio State University, 1900 Coffey Road, 444 Veterinary Medical Academic Building, Columbus, OH USA; 2 Department of Veterinary Biosciences, College of Veterinary Medicine, The Ohio State University, Columbus, OH USA

**Keywords:** Urothelial carcinoma, Receptor tyrosine kinase, Toceranib phosphate, Canine

## Abstract

**Background:**

Urothelial carcinoma (UC) accounts for > 90% of canine tumors occurring in the urinary bladder. Toceranib phosphate (TOC) is a multi-target receptor tyrosine kinase (RTK) inhibitor that exhibits activity against members of the split kinase family of RTKs. The purpose of this study was to evaluate primary UC tumors and UC cell lines for the expression and activation of VEGFR2, PDGFRα, PDGFRβ, and KIT to assess whether dysregulation of these RTKs may contribute to the observed biological activity of TOC.

**Results:**

Transcript for VEGFR2, PDGFRα, PDGFRβ, and KIT was detected in all UC tissue samples and UC cell lines. The Proteome Profiler™ Human Phospho-RTK Array Kit (R & D Systems) provided a platform to assess phosphorylation of 42 different RTKs in primary UC tumors and UC cell lines. Evidence of PDGFRα and PDGFRβ phosphorylation was present in only 11% or 33% of UC tumors, respectively, and 25% of UC cell lines. Treatment of UC cell lines with TOC had no significant impact on cell proliferation, including UC cell lines with evidence of PDGFRβ phosphorylation.

**Conclusions:**

Phosphorylation of several key RTKs targeted by TOC is present in a small subset of primary UC tumors and UC cell lines, suggesting that these RTKs do not exist in a state of continuous activation. These data suggest that activation of RTKs targeted by TOC is present in a small subset of UC tumors and UC cell lines and that treatment with TOC at physiologically relevant concentrations has no direct anti-proliferative effect on UC cells.

**Supplementary Information:**

The online version contains supplementary material available at 10.1186/s12917-021-03027-0.

## Introduction

Canine urothelial carcinoma (UC) represents 1.5% to 2% of all canine neoplasia and accounts for ≥ 90% of urinary bladder tumors in dogs. The majority [1, 2] of these tumors are classified on histopathology as high-grade papillary infiltrative tumors involving the bladder trigone with frequent extension into the urethra or the prostate in males. While the locally invasive behavior and trigonal location presents a significant clinical challenge with respect to local disease management in dogs, distant metastatic disease [2–4] is reported in approximately 15–20% of dogs at diagnosis and in ≥ 50% of dogs at death, with liver, lung and bone being the most frequent sites of spread.

Given the proclivity of UC tumors to develop in the trigone region of the urinary bladder, adequate surgical excision remains challenging and the majority of UC tumors are considered inoperable [5]. Alternatively, radiation therapy has been successfully utilized for the treatment of local disease; however, its use has been limited by a lack of durable response times and the development of late complications associated with pelvic irradiation (cystitis, urinary incontinence, chronic colitis) [6, 7]. Technological advancements in intensity-modulated and image-guided radiotherapy (IM/IGRT) [8, 9] have helped overcome several of the challenges related to the development of late, dose-limiting side effects and recent studies describe fractionated radiation protocols that resulted in an objective response rate of 61% with 100% of dogs experiencing clinical benefit (complete response, partial response, or stable disease) within 6 weeks of radiation therapy. Although these treatments have been shown to improve locoregional disease control and overall survival, most dogs with UC become refractory to treatment and succumb to local disease recurrence and/or metastasis, highlighting the need for effective systemic therapy. To this end, systemic medical therapy using a combination of cyclooxygenase inhibitors [10] and various chemotherapeutic agents (carboplatin, cisplatin, mitoxantrone, vinblastine, among others) [11–14] remains the mainstay of treatment for canine UC. While clinical symptoms often improve following systemic medical therapy, objective response rates remain poor, ranging from 18–36% and these are generally short lived with reported survival times ranging from 4–10 months [4]. As chemotherapy is expected to remain an important part of the clinical management of canine UC, novel and/or more effective therapies are urgently needed in order to alter the aggressive behavior of this cancer and improve outcomes for dogs with UC.

It is well-established that alterations in processes involved in tumor growth, progression and metastasis are mediated by ligand-receptor interactions involving receptor tyrosine kinases (RTKS) and downstream signaling molecules. Given the role of RTKs in the control of numerous cellular processes (particularly cell survival and proliferation) and the importance of aberrant RTK signaling in cancer, RTKs have emerged as important targets for the development of anticancer therapies. Toceranib phosphate (TOC) [15] (Palladia; Zoetis Animal Health, Madison, NJ, USA) is the first veterinary approved, multi-targeted receptor tyrosine kinase inhibitor that disrupts the function of several members of the split kinase RTK family, including vascular endothelial growth factor receptor 2 (VEGFR2), platelet derived growth factor receptors-alpha and -beta (PDGFR α/β), KIT, and Flt-3, among others [15–17]. While TOC originally received FDA approval for use in the treatment of canine mast cell tumors (MCT), subsequent studies [18] have demonstrated clinical activity in other tumor types including apocrine gland adenocarcinomas, thyroid carcinomas, gastrointestinal tumors, and head and neck carcinomas [19]. To this end, in a phase 1 study [17] in dogs with spontaneous malignancies, TOC demonstrated single agent activity against a variety of tumor types including UC, with 3 of 4 dogs with bladder UC treated with TOC experiencing stable disease for 10 weeks or greater. Gustafson et al. [20] reported on 37 dogs with bladder tumors that received TOC and observed a partial response in 6.7% of dogs, and stable disease in 80% of dogs for a median duration of 128.5 days; however, the biological basis for the observed responses to TOC in dogs with UC remains to be elucidated. Positive immunohistochemical staining for VEGFR2 and PDGFR-β has been documented in primary canine UC tumor tissues [21] and the expression of PDGFR-β in UCs was higher than that found in inflammatory cystitis tissues or normal bladder samples. While these data suggest a potential mechanism by which TOC may exert its activity in canine UC, a more comprehensive understanding of the pattern of RTK activation (phosphorylation), particularly those RTKs targeted by TOC, in canine UC tumors and UC cells is necessary. As such, the purpose of this study was to evaluate primary canine bladder UC tumors and established UC cell lines for the expression and activation of RTKs targeted by TOC and to evaluate the in vitro biological activity of TOC in canine UC cells.

## Results

### Sample demographics

Urothelial carcinoma (UC) tumor samples were collected from 9 canine patients that were presented to the Ohio State Veterinary Medical Center (OSU-VMC). The mean age was 10.3 years (range 7.3 to 13.0, median of 10.0 years). The patient breeds included the Scottish Terrier (*N* = 2), Maltese (*N* = 1), Shetland Sheepdog (*N* = 1), West Highland Terrier (*N* = 1), with the remainder being mixed breed dogs (*N* = 4). The tumor locations most frequently involved the bladder trigone (*N* = 4), the apex (*N* = 4), and the dorsal bladder wall (*N* = 1) with evidence of prostatic (*N* = 1) and urethral (*N* = 4) involvement on ultrasonographic imaging. None of the patients had evidence of locoregional metastasis at the time of diagnosis and none had evidence of distant metastasis. All of the patients were presented with clinical signs which included stranguria, dysuria, hematuria, and/or pollakiuria. All of the patients received therapy with a non-steroidal anti-inflammatory (NSAID; piroxicam, deracoxib, or meloxicam) with 6/9 patients receiving some form of cytotoxic chemotherapy. Additionally, 3/9 patients received therapy with toceranib phosphate (Table 1) and one of those patients also received vinblastine.

**Table 1:** Patient demographics

**Breed**	**Sex**	**Age (Yr)**	**Weight (kg)**	**Tumor Location**	**Regional Mets**	**Distant Mets**	**Chemo**	**TOC Therapy**
West Highland Terrier	S	7.3	6.5	Trigone	No	No	Yes	No
Scottish Terrier	S	10	9.0	Apex	No	No	Yes	No
Scottish Terrier	C	9	18.0	Apex	No	No	Yes	No
Maltese	C	8	5.2	Trigone, Urethra	No	No	Yes	No
Mixed	S	12	19.1	Apex, Urethra	No	No	No	No
Mixed	C	13	8.0	Apex	No	No	Yes	Yes
Mixed	S	11.2	18.5	Trigone, Urethra	No	No	No	Yes
Shetland Sheepdog	S	12.3	13.1	Dorsal Bladder Wall	No	No	No	Yes
Mixed	C	10	15.0	Trigone, Urethra, Prostate	No	No	Yes	No

The signalment of the patients from which nine primary tumor samples were collected is shown and included breed, sex, age, and weight. The presence of regional metastases (i.e. lymph nodes) and distant metastases (i.e. lung, bone, other intra-abdominal organs) were recorded. Whether patients received chemotherapy (not including toceranib phosphate) is denoted

*C* castrated, *S* Spayed

All tumor samples contained varying degrees of necrosis; however, this was limited to < 5% of the total tumor area in three of the patient samples and only 5–10% of the total tumor area in five of the samples. A single tumor sample was comprised of markedly more necrosis than other samples, with > 50% of the examined tissue consisting of necrotic debris (Additional file 1).

### RTK transcript expression in canine primary UC tumors and UC cell lines

**Fig. 1:**
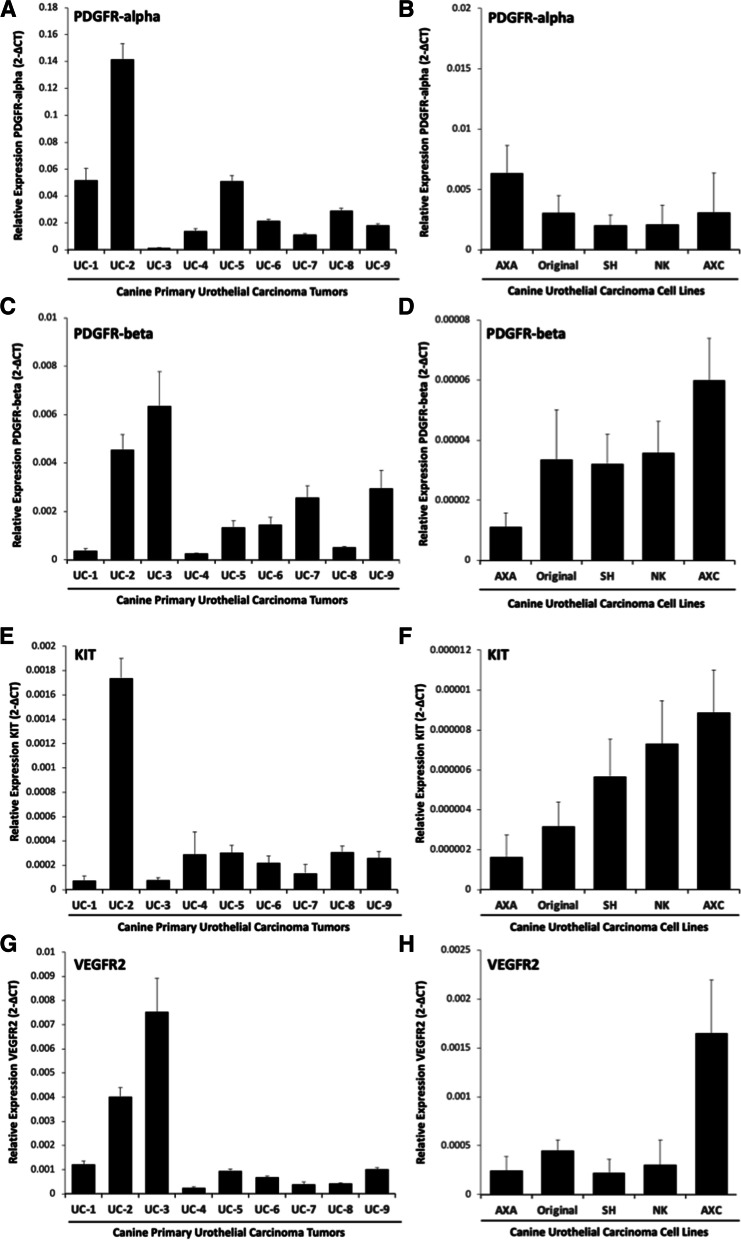
Receptor tyrosine kinase transcript expression in primary canine urothelial carcinoma tumor samples and primary urothelial carcinoma cell lines. Total RNA was isolated from primary canine urothelial carcinoma (UC) tumors (*N* = 9) and quantitative RT-PCR was performed to determine the relative expression of **A** PDGFRα, **C** PDGFRβ, **E** KIT, and **G** VEGFR2 transcript. All reactions were performed in triplicate. Data presented represent the mean, error bars = SD. Total RNA was isolated from canine urothelial carcinoma (UC) cell lines (*N* = 5) and quantitative RT-PCR was performed to determine the relative expression of **B** PDGFRα, **D** PDGFRβ, **F** KIT, and **H** VEGFR2 transcript. Three independent experiments were performed for each cell line and all reactions were performed in triplicate. Data presented represent the mean, error bars = SD from three experimental replicates

Real time PCR was performed to provide an initial assessment of mRNA transcript expression of the RTKs PDGFRa, PDGFRb, VEGFR2, and KIT in primary UC tumor samples (*N* = 9) and established canine UC cell lines (*N* = 5). Transcript for PDGFRa, PDGFRb, VEGFR2, and KIT was detectable in all of the primary UC tumor samples and UC cell lines (Fig. 1) although this was present at varying degrees. Consistent with previous studies demonstrating minimal immunohistochemical staining for KIT in UC tumor samples, we found that the expression levels of PDGFRα, PDGFRβ, and VEGFR2 transcript was higher in UC tumors and UC cell lines as compared to the relatively low basal expression of KIT in all samples.

### Phospho-receptor tyrosine kinase profiling in canine UC tumors and UC cell lines

**Fig. 2:**
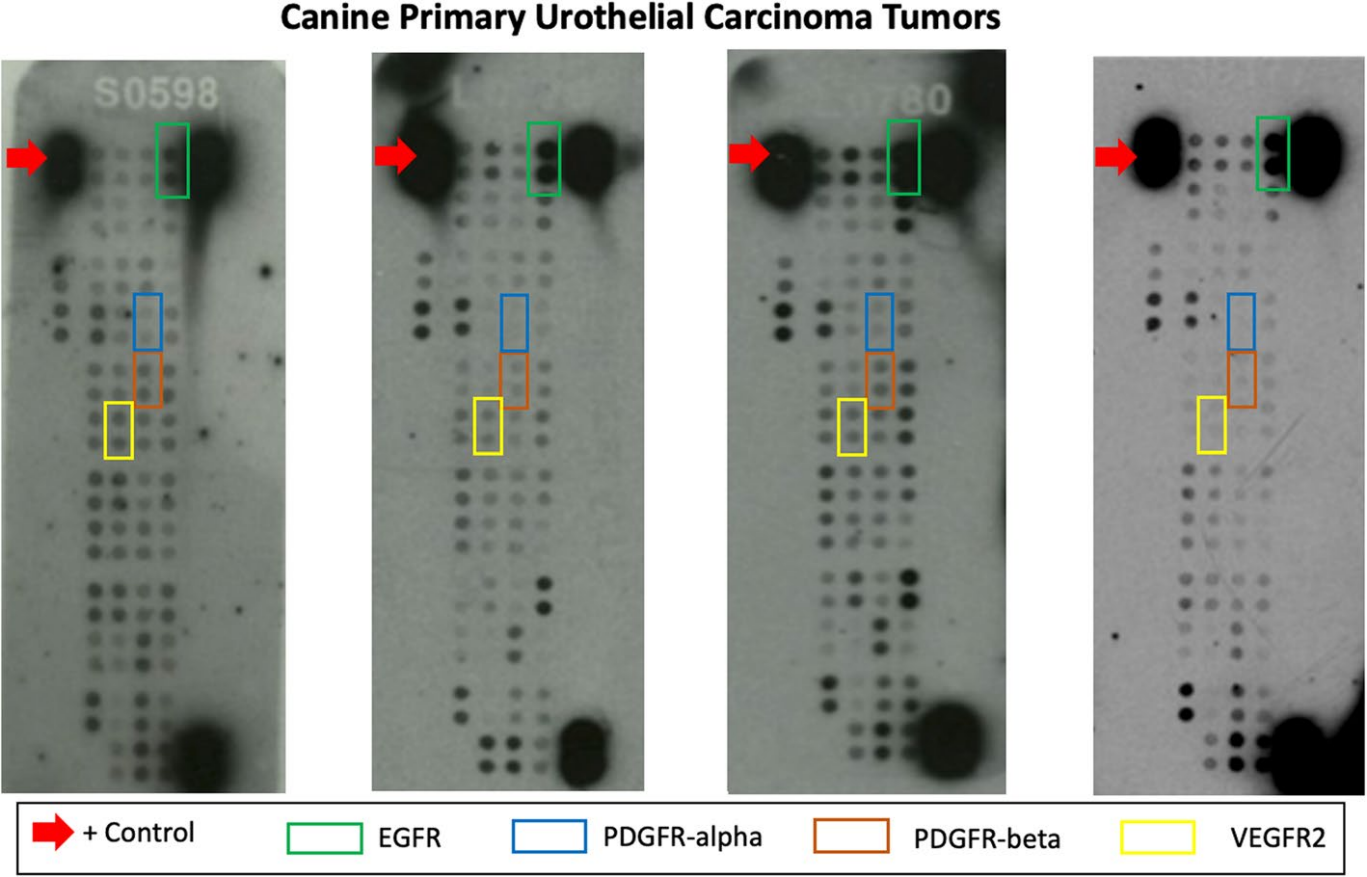
Phospho-RTK array profiling of primary canine UC tumors. Shown are representative examples of phosphoprotein arrays of primary UC tumors using the Proteome Profiler Human Phospho-RTK Array Kit. This assay allows for the simultaneous screening of 42 different RTKs. Determination of phosphorylation was based on comparison of capture antibody of interest to positive controls (red arrows) located on the periphery of the array. On these sample arrays, positive control EGFR has been identified for comparison. RTKs targeted by toceranib phosphate, PDGFRα, PDGFRβ, and VEGFR2 are indicated in the figure legend

**Fig. 3:**
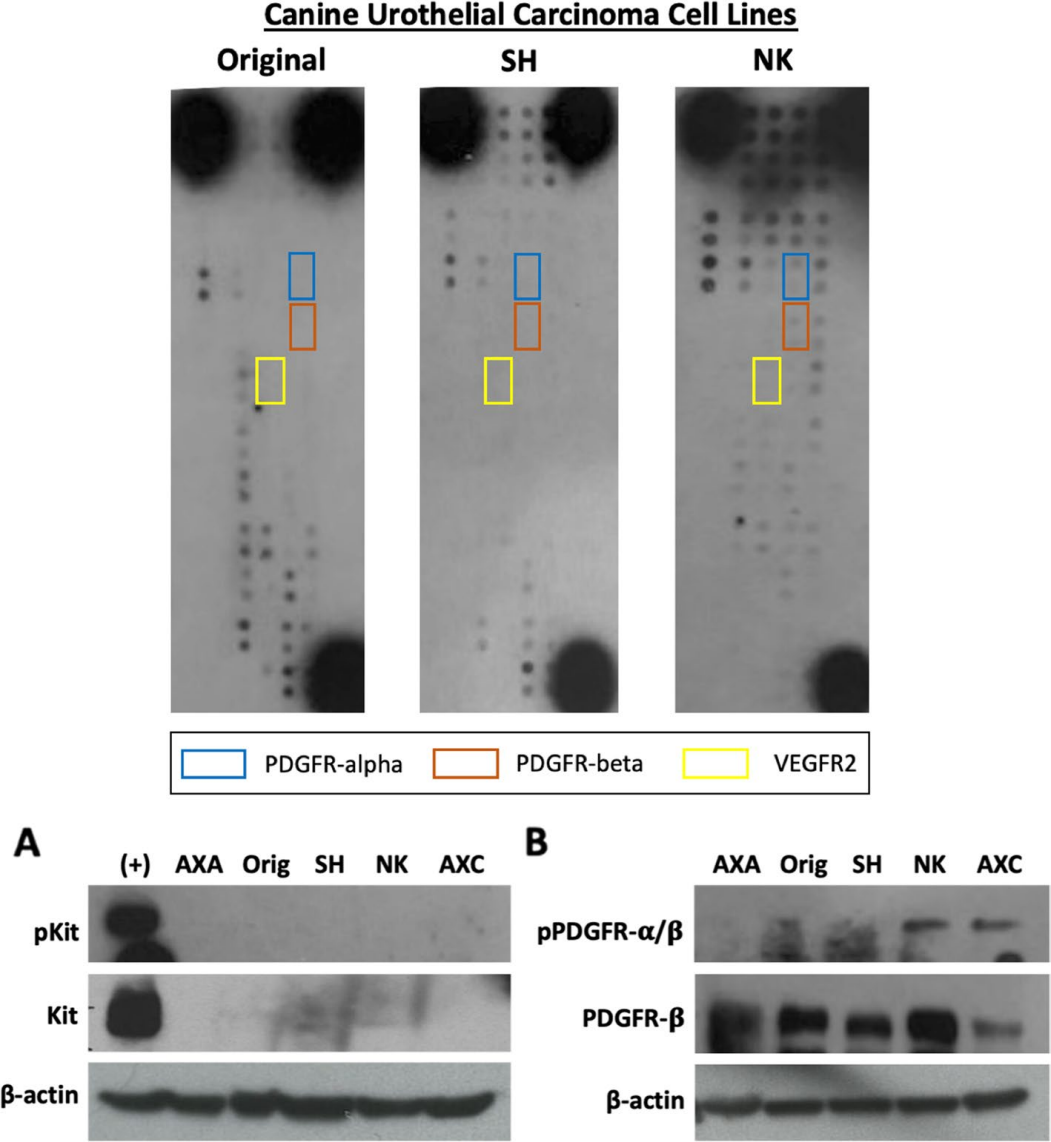
Western Blots for detection of pKit, total KIT, p-PDGFRα, and p-PDGFRβ and total PDGFRβ. Upper panel: Canine UC cells were serum starved for 2 h and protein lysates were generated. Relative phosphorylation of RTKs was assessed using the Proteome Profiler Human Phospho-RTK Array Kit. Shown are representative examples of phosphoprotein arrays of canine UC cell lines. RTKs targeted by toceranib phosphate, PDGFRα, PDGFRβ, and VEGFR2 are indicated in the figure legend. Lower panel: Canine UC cells were serum starved for 2 h and protein lysates were generated. Protein was separated by SDS PAGE and western blotting for **A** p-KIT, total KIT, and β-actin or **B** p-PDGFRα/β, PDGFRβ, and β-actin was performed to validate findings of the phosphoprotein arrays. The C2 canine mastocytoma cell line (Lane 1) was used as a positive control for detection of p-KIT and total KIT

The Proteome Profiler™ Human Phospho-RTK Array Kit was used to evaluate the phosphorylation and activation of cell surface RTKs in primary UC tumor samples and established UC cell lines. Representative examples of the phospho-RTK array are shown in Figs. 2 and 3 (uncropped arrays available in supplemental figure 1). A summary of the array results and observed differences in RTK phosphorylation between primary UC tumor samples and UC cell lines is provided in Table 2. Unsurprisingly, greater than 85% of primary UC tumors demonstrated phosphorylation of EGFR (100%) and ErbB2 (89%) which is concordant with prior studies suggesting that overexpression of ErbB2 and EGFR is a common event in this cancer [22, 23] and that alterations in ErbB2-mediatied [24] signaling pathways drive the proliferative phenotype in UC cells. In addition, we found that UC tumors showed phosphorylation of insulin receptor (78%), ROR1 (89%), ROR2 (89%), Tie-1 (100%), and Tie-2 (78%), with members of the FGFR family of receptors occurring less frequently (FGFR1 44%, FGFR2 44%, FGFR3 44%, and FGFR4 22%). Of the primary tumors evaluated, phosphorylation of PDGFRα and PDGFRβ was present in only 1/9 (11%) and 3/9 (33%) of tumors, respectively. Similarly, phosphorylation of VEGFR family members was present at varying degrees in 22% (VEGFR1), 33% (VEGFR2) and 56% (VEGFR3) of UC tumors. Lastly, phosphorylation of other RTKs targeted by toceranib including KIT (33%) and Flt-3 (22%) was observed in a relatively small percentage of tumor samples.

**Table 2:** Phospho-RTK profiling results for canine primary urothelial carcinoma tumor samples and urothelial carcinoma cell lines

**Receptor tyrosine kinase**	**Urothelial carcinoma tumors n (%)**	**Urothelial carcinoma cell lines n (%)**
PDGFRa	1 (11%)	1 (25%)
PDGFRb	3 (33%)	1 (25%)
KIT	3 (33%)	1 (25%)
Flt-3	2 (22%)	0 (0%)
M-CSF R	3 (33%)	1 (25%)
VEGFR1	2 (22%)	0 (0%)
VEGFR2	3 (33%)	0 (0%)
VEGFR3	5 (56%)	0 (0%)
EGFR	9 (100%)	4 (100%)
ErbB2	8 (89%)	4 (100%)
ErbB3	3 (33%)	2 (50%)
ErbB4	4 (44%)	1 (25%)
FGFR1	4 (44%)	1 (25%)
FGFR2	4 (44%)	1 (25%)
FGFR3	4 (44%)	1 (25%)
FGFR4	2 (22%)	0 (0%)
Insulin R	7 (78%)	2 (50%)
c-RET	3 (33%)	1 (25%)
ROR1	8 (89%)	4 (100%)
ROR2	8 (89%)	3 (75%)
Tie-1	9 (100%)	4 (100%)
Tie-2	7 (78%)	3 (75%)

The 22 most commonly observed phosphorylated receptor tyrosine kinases on the phospho-RTK Arrays are listed in the first column. The number of primary urothelial carcinoma tumors with the respective phosphorylated RTK is shown as a total and percentage of all tumors (*n* = 9). The number of primary urothelial carcinoma cell lines with the respective phosphorylated RTK is shown in the right column as a total and percentage of all cell lines (*n* = 5)

As the primary UC tumor samples evaluated in this study were not microdissected, it is therefore possible that these samples contain varying degrees of UC tumor cells and associated stromal and immune cells within the tumor microenvironment. To evaluate and compare phosphorylation of RTK specific to UC cells, we profiled phospho-RTK expression in established canine UC cell lines. Interestingly, the overall profile of phospho-RTK expression of UC cell lines was largely equivalent to that observed in the primary UC tumor samples with several exceptions (Table 2). Similar to our findings in primary UC tumor samples, EGFR and ERbB2 phosphorylation was observed in all cell lines. In contrast to UC tumor specimens, phosphorylation of members of the FGFR family of RTKs was lower in UC cell lines (FGFR1 25%, FGFR2 25%, FGFR3 25%, and FGFR4 0%) which may reflect the presence of stromal cells within the primary tumor samples evaluated. UC cell lines showed evidence of phosphorylation of insulin receptor (50%), ROR1 (100%), ROR2 (75%), Tie-1 (100%), and Tie-2 (75%). Phosphorylation of PDGFRα and PDGFRβ was present in only 25% of UC cell lines and 0% of cell lines demonstrated phosphorylation of VEGFR1, VEGFR2, or VEGFR3. Similarly, phosphorylation of VEGFR family members was present at varying degrees in 22% (VEGFR1), 33% (VEGFR2) and 56% (VEGFR3) of UC tumors. Lastly, phosphorylation of key RTKs targeted by TOC including KIT (25%) and Flt-3 (0%) was observed in UC cell lines.

Western blotting was performed to confirm and validate the findings of the phospho-RTK array in canine UC cell lines. As shown in Fig. 3, phosphorylation of KIT was not detected in any of the UC cell lines evaluated. While all UC cell lines expressed total PDGFβ protein, evidence of phosphorylated PDGFRα/β was detected in only two of the five (40%) UC cell lines evaluated (see supplemental Figure 1 for uncropped western blots).

### Biological activity of toceranib phosphate on canine urothelial carcinoma cell lines

**Fig. 4:**
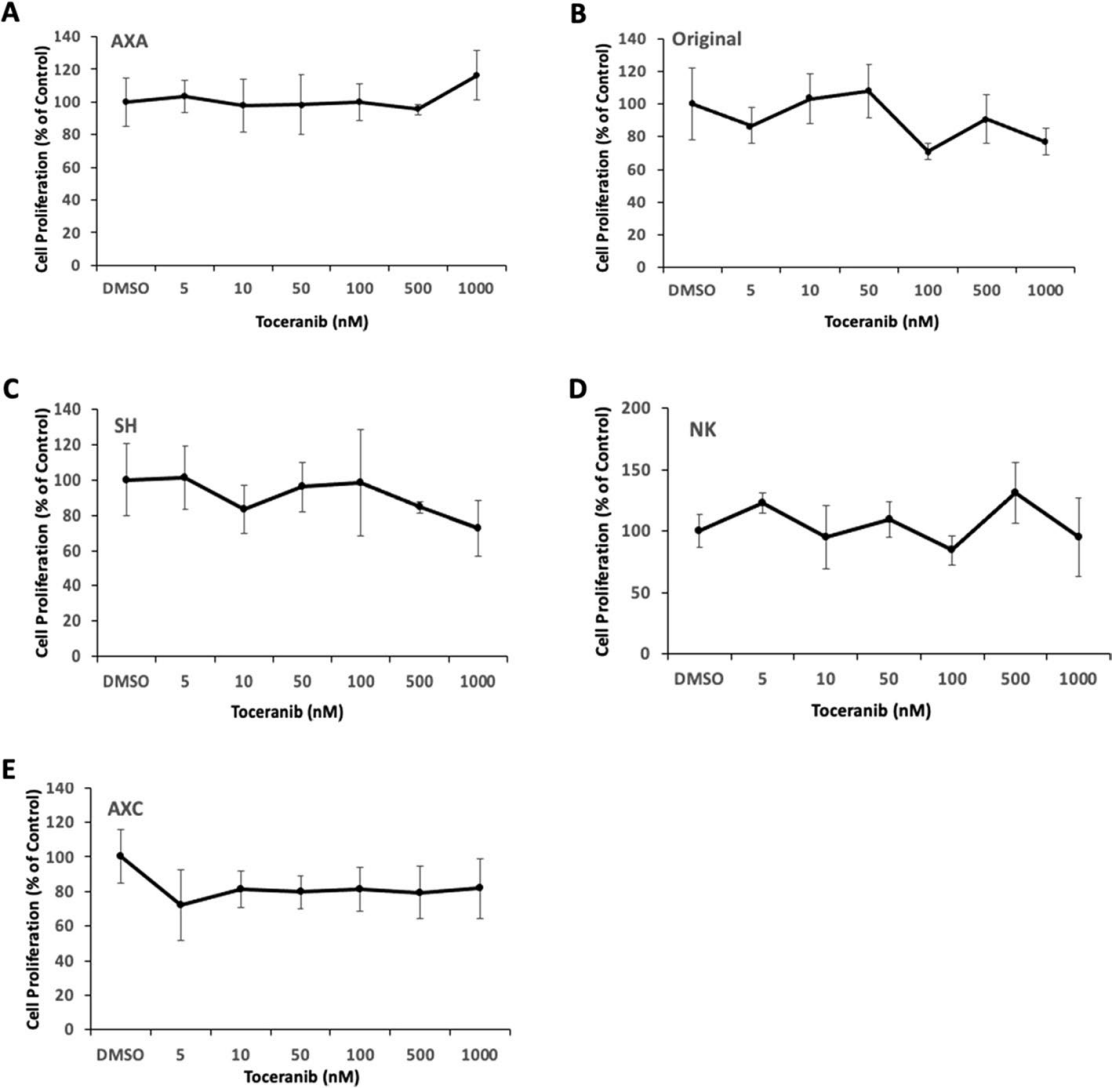
Toceranib phosphate has no direct effect on cell proliferation in canine urothelial carcinoma (UC) cell lines. Canine UC cell lines **A** AXA, **B** Original (Orig), **C** SH, **D** NK and **E** AXC were treated with DMSO or increasing concentrations of toceranib phosphate for 48 h and cell proliferation was assessed using the CyQUANT assay kit. Values listed are a percentage of DMSO control. Experiments were performed in triplicate and repeated three times. Data presented represent the mean, error bars = SD

To assess the direct in vitro activity of toceranib phosphate on UC cell line growth and proliferation, canine UC cell lines were treated with increasing doses of toceranib phosphate to determine whether toceranib phosphate was capable of inhibiting the proliferative capacity of UC cells. Cells were treated with 5, 10, 50, 100, 500, or 1000 nM toceranib for 48 h and proliferation was determined using the CyQUANT assay. As shown in Fig. 4, no significant differences in cell proliferation were detected in any of the UC cell lines evaluated even at relatively high nanomolar concentrations (up to 1 µM) of toceranib phosphate.

## Discussion

Urothelial carcinoma (UC) accounts for over 90% of canine malignancies occurring in the urinary bladder and despite aggressive therapies including partial cystectomy, radiotherapy, and systemic chemotherapy, long term outcomes for dogs remains poor. While clinical signs often improve following systemic medical therapy, objective response rates are noted in 18–36% [4, 25] of treated patients and duration of response is generally short lived. In other cancers, an increased understanding of tumor biology has provided support for the notion that selective targeting of cell signaling pathways may improve the efficacy of therapy. More specifically, inhibition of receptor tyrosine kinases (RTKs) represents a targeted strategy to block tumor cell proliferation, invasion and survival. The purpose of the current study was to evaluate primary UC tumors and available UC cells lines for expression of RTK transcript and activation (phosphorylation) of RTKs targeted by toceranib phosphate (TOC) to better understand the biological basis for the observed responses of this tumor to TOC.

While transcript for PDGFRα, PDGFRβ, VEGFR2 and KIT was detected in all tumor samples and UC cell lines, the pattern of expression was variable. Furthermore, while mRNA was present in UC tumor tissues and UC cell lines, the presence of RTK phosphorylation for these RTKs was present in only 11–33% of tumor specimens and UC cell lines suggesting that activation of these RTKs is present in only a small subset of UC cells. Importantly, the absence of RTK pathway activation for PDGFRα, PDGFRβ, VEGFR2 and KIT in the majority of UC tumors and UC cell lines suggests that signaling through these pathways is unlikely to drive the aggressive behavior of UC cells. Prior studies [21] have demonstrated increased expression of total PDGFRβ protein in UC tumors compared to tissues from dogs with normal or polyploid cystitis; however, in these studies the phosphorylation status was not evaluated. In the current study, we found that phosphorylation of PDGFRβ was present in only 33% of UC tumor tissues and 25% of UC cell lines. We confirmed this finding using western blotting, which showed that only two of the five UC cell lines had evidence of phosphorylation of PDGFRβ. Collectively, these findings indicate that activation of RTKs targeted by TOC is only present in a small subset of UC tumor cells and suggest that therapeutic targeting these RTKs is unlikely to substantially alter the aggressive behavior of UC cells in the majority of patients.

Of note, evidence of RTK phosphorylation was variable in primary UC tumor samples evaluated in this study. This variability may be attributed to tumor tissue heterogeneity as the samples were not microdissected and thus included tumor cells, associated stromal cells within the tumor microenvironment and to a lesser degree, necrosis. The presence of cell types within the tumor microenvironment may confer presence of growth factors and other cytokines that influence the phosphorylation status of RTKs which may otherwise not be activated in UC cells. Additionally, primary tumor tissues were collected from patients that received various chemotherapy treatments prior to sample procurement. It is therefore possible that prior treatment with intravenous chemotherapeutic agents, toceranib phosphate, and/or NSAIDs may have affected the expression and phosphorylation profile of the RTKs of interest in this study population. To further dissect the potential influence of RTK pathway activation on UC cell behavior, we profiled RTK activation in established UC cell lines which represent a model system without the confounding influence of tumor stromal and/or immune cells. Importantly, the cells were serum starved for 2 h in order to eliminate the potential for endogenous growth factor influence on the phosphorylation status of the RTKs. We observed that in the primary tumor samples evaluated, the profile of RTK activation in UC cells lines was similar to that found in tumors with evidence of PDGFRα and PDGFRβ phosphorylation present in only 25% of UC cell lines evaluated. While our results from the phospho-RTK arrays showed moderate phosphorylation of the RTKs of interest, it is important to note the possibility of false negative results. The phospho-RTK array used in the present study was originally designed to detect a wide variety of human phosphorylated RTKs and utilizes optimized human antibodies. The array has not been fully validated in canine tissues and therefore, may underestimate the true phosphorylation status of the primary tumors due to a lack of antibody cross-reactivity.

Importantly, we evaluated the direct effect of TOC on cell proliferation in UC cell lines showing variable levels of PDGFR activation and found that treatment with TOC did not result in a dose-dependent decrease in cell viability. Small molecule inhibitors such as TOC frequently demonstrate biological activity in cancer cell lines at low nanomolar concentrations and this correlates with physiologically relevant pharmacokinetic profiles for dogs receiving TOC [26]. We failed to detect a significant difference in UC cell growth inhibition following treatment with relatively high nanomolar concentrations of TOC, indicating that TOC does not directly alter the proliferative behavior of UC cell lines in vitro even at doses exceeding that routinely used in clinical practice. All cell proliferation assays were conducted in media containing 10% fetal bovine serum; therefore, it is possible that RTK stimulation or activation by exogenous growth factors within the media may have contributed, in part, to the negligible effect of TOC treatment on UC cell line proliferation. These data are concordant with our RTK profiling results which show that RTKs targeted by TOC infrequently show evidence of phosphorylation and that inhibition of these RTKs are unlikely to have a substantial activity on UC cell proliferation or clinically meaningful benefit.

Clinical benefit has been described in dogs with UC receiving TOC and a non-steroidal anti-inflammatory drug (piroxicam) with the majority of dogs (80%) in this study experiencing stable disease [20]. In light of our data demonstrating that TOC does not appear to have a significant direct impact on UC cell line proliferation this suggests that reported clinical benefit seen in UC patients receiving TOC may be due, in part to off-target effects or inhibition of RTK activity in cells present within the tumor microenvironment. Recent data [27] suggest that TOC may have indirect effects on tumor biology by modulating the tumor microenvironment via inhibition of angiogenesis and/or altering the activity of infiltrating regulatory T cells. Indeed, the BRAF^V595E^ mutation which is present in 75–87% of canine UC tumors [28] has been shown to be associated with tumor-produced CCL17 and regulatory T cell infiltration in dogs with UC highlighting the potential influence of the tumor immune environment on UC tumor behavior. The observed biological activity of TOC in other canine carcinomas [29, 30] has been suggested to be due to indirect anti-tumor effects of TOC on cellular components existing within the tumor microenvironment; therefore, it is possible that TOC may exhibit some anti-tumor behavior in canine UC tumors that is a result of indirect targeting of cellular components of the tumor immune environment and warrants further investigation.

## Conclusion

The present study provides an initial characterization of the activation and phosphorylation of RTKs in primary canine UC tumors and UC cell lines. We observed that phosphorylation of several key RTKs targeted by TOC is present in a small subset of primary UC tumor samples and UC cell lines, suggesting that many of these RTKs do not exist in a state of continuous activation in UC tumors. Treatment of UC cell lines with TOC had no significant impact on cell proliferation, including those with evidence of PDGFRβ phosphorylation. Furthermore, UC cell proliferation was not significantly altered following treatment of UC cell lines with TOC in vitro*,* even at micromolar concentrations. Collectively, these data demonstrate that activation of RTKs targeted by TOC is present in a small subset of UC tumors and UC cell lines and that treatment with TOC at physiologically relevant concentrations has no direct anti-proliferative effect on UC cells.

## Methods

### Cell lines and primary tumor samples

The canine urothelial carcinoma (UC) cell lines K9TCC-PU-AXA, K9TCC-PU-AXC, K9TCC-PU-NK, K9TCC-PU-Original, and K9TCC-PU-SH were generously provided by Dr. Deborah Knapp (Purdue University, IN, USA). All cell lines were previously established and validated [31, 32]. Cells were not subjected to canine species verification testing; however, morphology under light microscopy and growth kinetics remained consistent throughout the experiments. The cells were cultured in Dulbecco’s Modified Eagle Medium (Gibco™ Life Technologies, Grand Island, NY, USA). Media was supplemented with 10% fetal bovine serum (FBS) (Cat # 12483-020, Gibco™ Life Technologies), 1% GlutaMAX™ (Cat #1963762, Gibco™ Life Technologies), 1% Antibiotic–Antimycotic (Cat #15240-062, Gibco™ Life Technologies), 1% HEPES (4-(2-hydroxyethyl)-1-piperazineethanesulfonic acid), 1% NEAA (non-essential amino acids), and 1% sodium pyruvate (all media supplements from Gibco™ Life Technologies). The canine mastocytoma cell line C2 (*KIT* ITD mutation in the JM domain, generously provided by Dr. Warren Gold, Cardiovascular Research Institute, University of California – San Francisco, CA, USA) served as a positive control cell line for toceranib phosphate drug treatment assays and was maintained in Roswell Park Memorial Institute (RPMI) 1640 medium (Gibco™ Life Technologies) supplemented with 10% fetal bovine serum, non-essential amino acids, sodium pyruvate, antibiotic–antimycotic, GlutaMAX™, and HEPES. All cell lines were cultured in a humidified incubator containing 5% CO_2_ at 37 °C. The cells were routinely tested for mycoplasma and treated with Plasmocin (Cat.#ant-mpt-1, Invivogen) as necessary.

### Primary urothelial carcinoma tumor samples

Primary canine UC tumor samples (*N* = 9) were collected from clinical cases treated at The Ohio State University Veterinary Medical Center (OSU-VMC). Owner consent for tissue collection was obtained in all cases in accordance with established hospital protocols and approved Institutional Animal Care and Use Committee protocols (IACUC #2010A0015). Tissue collections were performed by the OSU-VMC Blue Buffalo Clinical Trials Office and Veterinary Clinical Research Shared Resource. Fresh tumors collected at the time of surgery or euthanasia were flash frozen in liquid nitrogen and placed in formalin and processed for routine paraffin embedding for histopathology. Briefly, sections were routinely sectioned at 4–5 µm thickness and stained with hematoxylin and eosin (H&E) on a Leica ST5020 autostainer (Leica Biosystems, Buffalo Grove, IL) using a routine and quality-controlled protocol. All tumor samples were confirmed to be urothelial carcinoma by board certified veterinary anatomic pathologists at OSU-VMC.

Sections were evaluated using a semi-quantitative assessment of the approximate percentage of total tumor area comprised of necrotic debris using a digital grid with a total size of 3.2 × 2.3 mm and each square of the grid measuring 0.2 × 0.2 mm applied via Olympus CellSens Imaging Software. Semi-quantitative methods were developed in consultation with a board-certified veterinary comparative pathologist to assess the approximate percentage of total tumor area comprised of necrotic debris with subcategories < 5%, 5–10%, > 10%, and > 50% of total tumor. This was a pilot study with the intent to characterize the expression and phosphorylation of receptor tyrosine kinases in primary canine UC tumors and UC cell lines, so no power calculation was made.

### RNA isolation, cDNA synthesis, and qRT-PCR

Previously published [29] primers for canine VEGFR2, PDGFRα, PDGFRβ, KIT and GAPDH were evaluated and the primer efficiency was determined for each primer pair by constructing serial dilution curves and calculating the coefficient of determination (*R*^2^) and amplification efficiency. All primer sets demonstrated high amplification efficiency (96–98%) with a correlation coefficient (*R*^2^) ≥ 0.93 (Table 3). Primers were designed using Primer-BLAST and forward/reverse primers were designed to span an intron and detect all known splice variants for target genes evaluated [33]. All primers were tested for specificity by confirming the appropriate product amplicon size on standard agarose gel electrophoresis and sequencing the amplicons from a Real-Time quantitative PCR (qRT-PCR) reaction performed on normal control canine testes tissue. NormFinder alogorithm was used to determine the stability/variation value among 3 candidate housekeeping gene primer sets (GAPDH, 18S, β-Actin) and canine GAPDH was found to have the lowest stability value (GAPDH, V = 0.048; 18S, V = 0.166; β-Actin, V = 0.350); therefore, normalization was performed relative to GAPDH internal control [34, 35]. Total RNA was isolated from primary canine UC tumor tissues or UC cell lines using TRIzol reagent (Invitrogen™ Life Technologies) and column purified using the RNeasy Mini Kit (Qiagen, Germantown, MD, USA) according to manufacturer’s instructions. RNA quantification was carried out using a NanoDrop 2000 spectrophotometer (Thermo Fisher Scientific, Waltham, MA, USA) and cDNA was synthesized from 2 μg of total RNA using Superscript III (Invitrogen). Published primers for canine VEGFR2, PDGFRα, PDGFRβ, KIT and GAPDH [29] were used with Fast SYBR Green Master Mix (Applied Biosciences, Foster City, CA, USA) and Real-Time quantitative PCR (qRT-PCR) was performed using the Applied Biosystems StepOne Plus Detection System (Applied Biosystems). Relative mRNA expression levels of each RTK were determined using the comparative threshold cycle method [36] and normalized to the endogenous housekeeping gene GAPDH. All reactions were performed in triplicate and three independent experiments were carried out in UC cell lines. All reactions included no-template controls for each gene.

**Table 3:** List of reference genes and target genes used for RT-qPCR

Gene/pimers	Primer sequences	Amplicon size (bp)	Primer efficiency	Correlation coefficient (*R*^2^)
*Canine PDGFRA, NCBI Reference Sequence: XM_038685627.1*				
Canine PDGFRA 1917F	5’-GCTCTCATGTCGGAACTGAAG-3’	382	96.8%	0.93
Canine PDGFRA 2152R	5’-GTGTGCTGTCATCAGCAGG-3’			
*Canine PDGFRB, NCBI Reference Sequence: NM_001003382.1*				
Canine PDGFRB 2286F	5’-GACGAGTCAGTGGATTACGTG-3’	327	97.1%	0.93
Canine PDGFRB 2612R	5’-GTCTCTCATGATGTCACGAGCCAG-3’			
*Canine KIT, NCBI Reference Sequence: NM_001003181.1*				
Canine KIT 260F	5’-GAGAACACACACAACGAATG-3’	183	97.6%	0.97
Canine KIT 442R	5’-GCAGCGGACCAGCGTATCATTG-3’			
*Canine KDR, NCBI Reference Sequence: NM_001048024.1*				
Canine VEGFR2 1537F	5’-GTAAGTACCCTTGTTATCCAAGCAGCC-3’	192	98.3%	0.96
Canine VEGFR2 1728R	5’-CGTAGTTCTGTCTGCAGTGCACCAC-3’			
*Canine GAPDH, NCBI Reference Sequence: NM_001003142.2*				
Canine GAPDH 588F	5’-GTCCATGCCATCACTGCCACCCAG-3’	193	97.4	0.96
Canine GAPDH 780R	5’-CTGATACATTGGGGGTGGGGACAC-3’			

The table shows a list of primers utilized in performing real time quantitative PCR. Listed are sequences for each primer/ gene as well as their size, *R*^2^, and listed primer efficiency by the supplier

### Protein lysate preparation and phosphoprotein arrays

The Proteome Profiler™ Human Phospho-RTK Array Kit (R&D Systems, Minneapolis, MN, USA) was used to assess relative phosphorylation of 42 different RTKs in primary UC tumor tissue samples and UC cell lines. Briefly, flash frozen tumor samples were pulverized in a frozen mortar and the resulting powder was resuspended in liquid nitrogen and transferred to a 1.5 mL microcentrifuge tube. Upon evaporation of the liquid nitrogen, samples were resuspended in 100–200 μL of complete tissue lysis buffer. Complete tissue lysis buffer was prepared per manufacturer’s instructions using 2.5 mL of Lysis Buffer-17 supplemented with phosphatase and protease inhibitors (2 mg/mL of aprotinin, 2 mg/mL leupeptin, and 5 mg/mL pepstatin A). Samples were rocked at 4 °C for 1 h, centrifuged for 15 min at 15,000 rpm at 4 °C, and the supernatants collected. Prior to collection, primary UC cell lines were serum starved for 2 h, washed twice with 1X Dulbecco’s Phosphate Buffered Saline (Gibco™ Life Technologies), pelleted, and protein lysates were prepared as described above. Extracted protein concentration was quantified using the Bradford assay BioRad Reagent (Cat # 5000006, BioRad, Hercules, CA, USA) and 100 µg total protein lysate was added to each RTK array and developed according to manufacturer’s directions.

### Immunoblotting

Canine UC cell lines (K9TCC-PU-AXA, K9TCC-PU-AXC, K9TCC-PU-NK, K9TCC-PU-Original, and K9TCC-PU-SH) were serum starved for 2 h and protein lysates were generated for western blotting, as described previously [37]. 100 µg protein was separated by SDS-PAGE on 4–20% Mini-PROTEAN® TGX™ precast protein gels (Cat #4561093, BioRad) and transferred overnight at 4 °C and 20 mV to PVDF membranes. Membranes were blocked in TBS-T containing 5% bovine serum albumin for 1 h at room temperature and incubated overnight with p-PDGFRα/β (C43E9, Tyr849/Tyr857, Cat #3170, Cell Signaling Technology, 1:500 dilution), anti-PDGFRβ antibody (Y92, Cat #32570, Abcam, 1:1000 dilution), p-KIT (Ty719, Cat #3391, Cell Signaling Technology, 1:1000 dilution), and KIT (PC34, Cat #961–976, Calbiochem, 1:500 dilution) at 4 °C. Membranes were incubated in horseradish peroxidase linked anti-rabbit or anti-mouse secondary antibody (Cat #7074, Cat #7076, Cell Signaling Technology), washed and exposed to substrate (SuperSignal West Dura Extended Duration Substrate, Pierce, Rockford, Illinois) Membranes were stripped using Restore™ Western Blot Stripping Buffer (Cat #21059, ThermoFisher Scientific), washed, blocked, and re-probed for β-actin (8H10D10, Cat #3700, Cell Signaling Technology).

### Cell proliferation

The effect of toceranib phosphate on canine UC cell line viability was assessed with the CyQUANT® Cell Proliferation Assay Kit (Molecular Probes, Eugene, OR, USA), as has been described previously [38]. 5 × 10^3^ cells were seeded in 96-well plates and incubated in media overnight. Cells were treated with 0.1% DMSO (control) or increasing concentrations of toceranib phosphate (0.005 µM – 1 µM) for 48 h. The canine C2 mastocytoma cell line which exhibits a predictable dose–response curve following treatment with toceranib phosphate was included in all assays as a positive control for cell proliferation assays. Briefly, 1 × 10^4^ C2 cells were seeded in 96-well plates, incubated overnight in complete medium overnight and treated with 0.1% DMSO (control) or increasing concentrations of toceranib phosphate (0.005 µM – 1 µM) for 48 h at 37 °C. Media was removed at 48 h and plates were frozen overnight at -80 °C. Fluorescence was measured with the UV Spectromax M2 plate reader (Molecular Devices, Sunnyvale, CA, USA) with excitation at 485 nm and emission detection at 530 nm. Cell proliferation was calculated as a percentage of the control DMSO wells. All treatments were evaluated in triplicate in three independent experiments.

### Statistics

All experiments were performed in triplicate and repeated three independent times for UC cell lines. Data were presented as mean plus or minus standard deviation. All qRT-PCR data was normalized to endogenous control (GAPDH) and the ∆∆ Ct method [36] was used to compare relative mRNA expression in UC tumor samples and UC cell lines. Group comparisons in the CyQUANT Cell Proliferation assays were analyzed by one-way ANOVA. Values of *p* < 0.05 were considered statistically significant.

## Supplementary Information

**Additional file 1:** Semi- quantitative Assessment of Percentage of Tumor Necrosis. **Supplemental Table 1.** summarizes the findings and degree of necrosis observed in the primary UC tumor samples. A single tumor sample was comprised of markedly more necrosis than the remaining samples, with >50% of examined tissue consisting of necrotic debris.

**Additional file 2: Figure S1.** Uncropped RTK arrays and western blots. (Fig. 3A) Phospho-RTK arrays on primary UC tumors. (Fig. 3A) Phospho-RTK arrays on canine UC cell lines. (Fig. 3B) Canine UC cell lines were serum starved for 2 h and protein lysates were generated. Protein was separated by SDS PAGE and western blotting for p-KIT, total KIT, and β-actin or p-PDGFRα/β, PDGFRβ, and β-actin was performed to validate findings of the phosphoprotein arrays. The C2 canine mastocytoma cell line (Lane 1) was used as a positive control for detection of p-KIT and total KIT. Red arrows indicate band corresponding to p-KIT, KIT, p-PDGFRα/β, PDGFRβ, or β-actin.

## Data Availability

The datasets used and/or analyzed during the current study are available as supplementary information or are available from the corresponding author on reasonable request.

## Abbreviations

VEGFR2: Vascular endothelial growth factor receptor 2
PDGFRα: Platelet derived growth factor receptor alpha
PDGFRβ: Platelet derived growth factor receptor beta
KIT: KIT proto-oncogene receptor tyrosine kinase
RTK: Receptor tyrosine kinase
UC: Urothelial carcinoma
IMRT: Intensity-modulated radiotherapy
IGRT: Image-guided radiotherapy
TOC: Toceranib phosphate
qRT-PCR: Real-time quantitative PCR
