# Supplementary figures and images for: Characterization of receptor tyrosine kinase activation and biological activity of toceranib phosphate in canine urothelial carcinoma cell lines
Source: BMC Vet Res. 2021 Oct 2;17:320. doi: 10.1186/s12917-021-03027-0 (PMC8487586; doi:10.1186/s12917-021-03027-0)

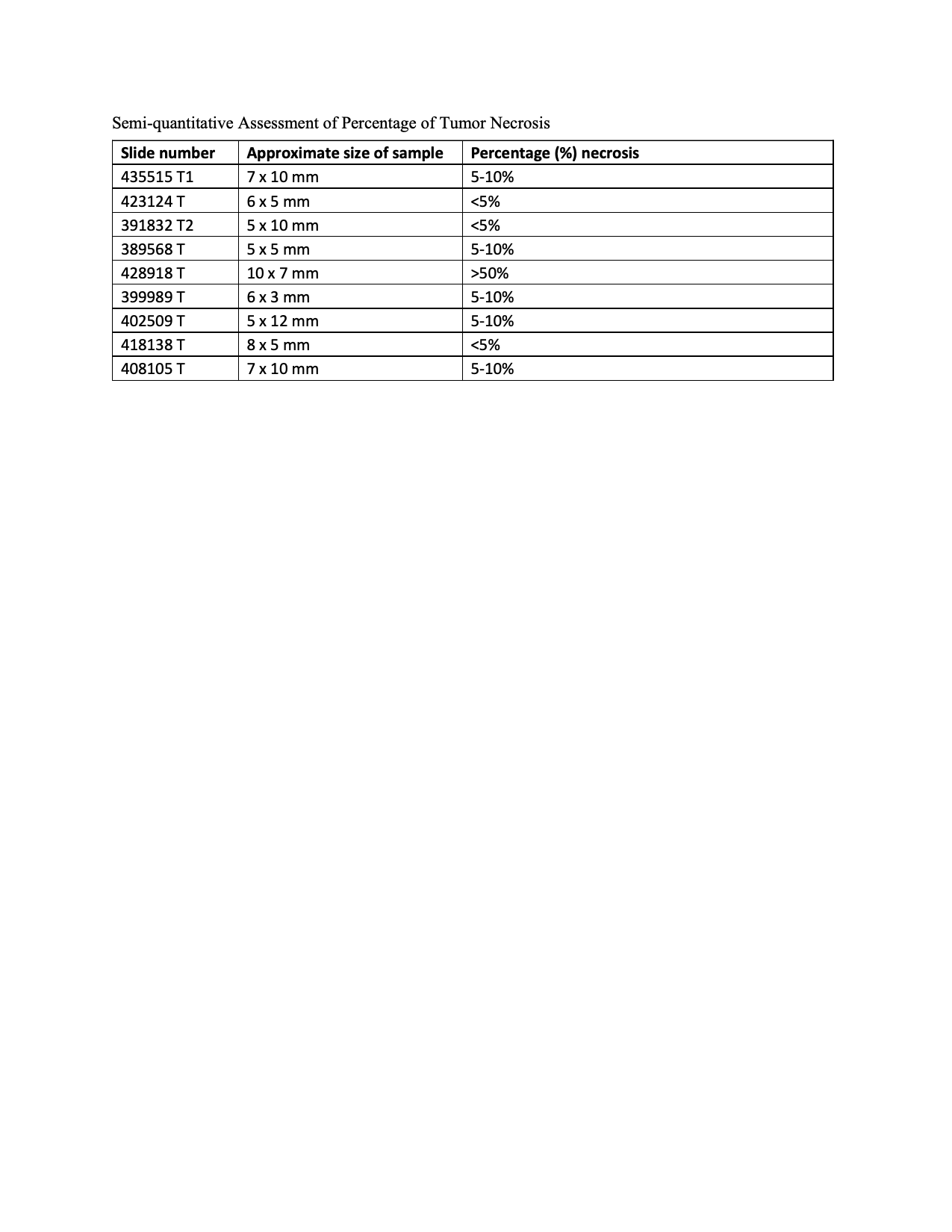

Supplement: Supplementary file 1 — Additional file 1: Semi- quantitative Assessment of Percentage of Tumor Necrosis. Supplemental Table 1. summarizes the findings and degree of necrosis observed in the primary UC tumor samples. A single tumor sample was comprised of markedly more necrosis than the remaining samples, with >50% of examined tissue consisting of necrotic debris. [file 12917_2021_3027_MOESM1_ESM.tiff]

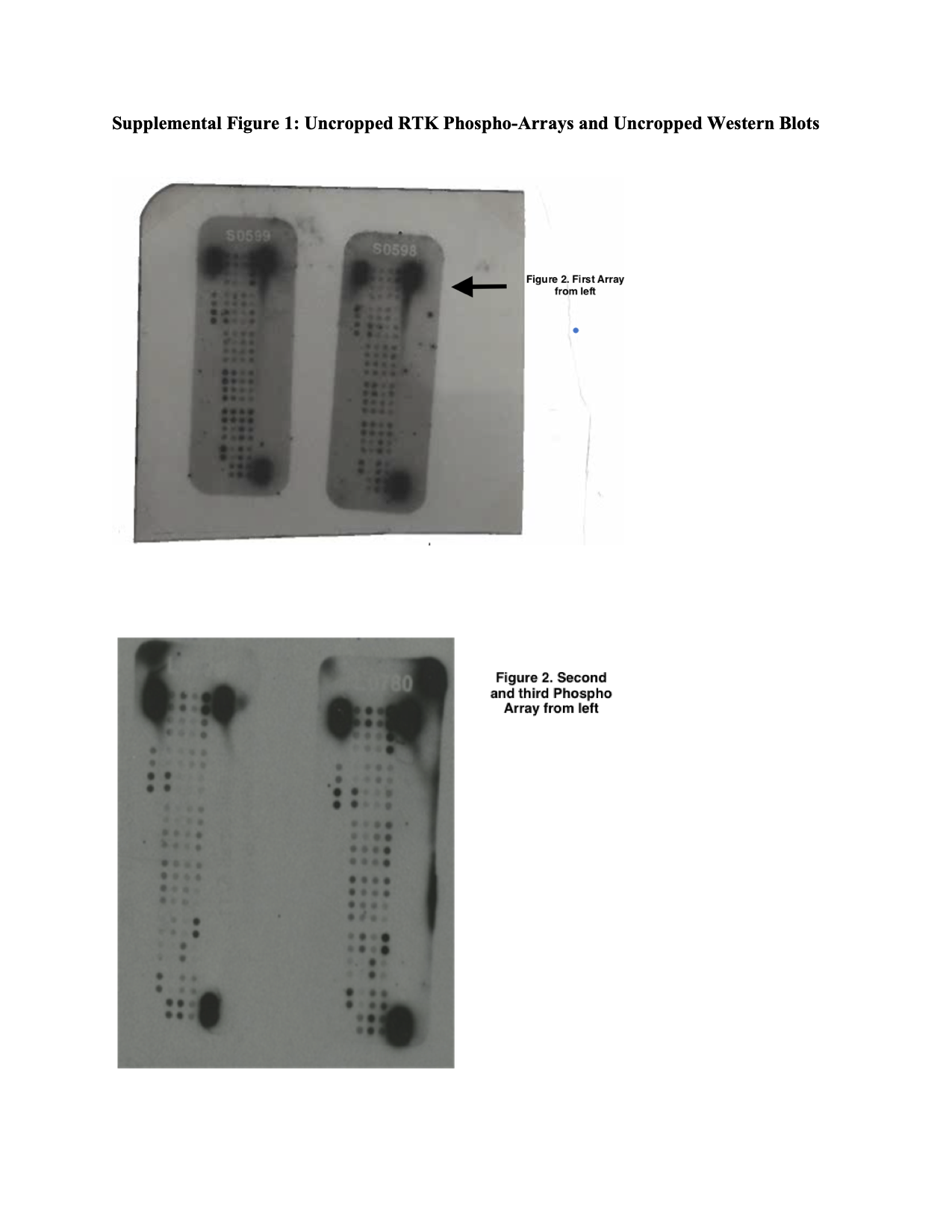

Supplement: Supplementary file 2 — Additional file 2: Figure S1. Uncropped RTK arrays and western blots. (Fig. 3A) Phospho-RTK arrays on primary UC tumors. (Fig. 3A) Phospho-RTK arrays on canine UC cell lines. (Fig. 3B) Canine UC cell lines were serum starved for 2 h and protein lysates were generated. Protein was separated by SDS PAGE and western blotting for p-KIT, total KIT, and β-actin or p-PDGFRα/β, PDGFRβ, and β-actin was performed to validate findings of the phosphoprotein arrays. The C2 canine mastocytoma cell line (Lane 1) was used as a positive control for detection of p-KIT and total KIT. Red arrows indicate band corresponding to p-KIT, KIT, p-PDGFRα/β, PDGFRβ, or β-actin. [file 12917_2021_3027_MOESM2_ESM.tiff]
